# Practical approaches to labelling terminal alkynes with deuterium

**DOI:** 10.1002/jlcr.3963

**Published:** 2022-02-02

**Authors:** Melanie Y. T. Chan, Arbab Anwar, William J. S. Lockley

**Affiliations:** ^1^ Department of Chemistry, Faculty of Engineering and Physical Sciences University of Surrey Guildford UK

**Keywords:** ^2^H, base‐catalysis, D, deuterium, HIE, isotope effect, isotope exchange, silver perchlorate, silver‐catalysis, terminal alkyne

## Abstract

Base catalysed exchange with sodium hydroxide, calcium oxide or *N*,*N*,*N*,*N*‐tetramethylguanidine in deuterium oxide is a viable procedure for the preparation of terminally deuterated alkynes for those alkynes stable to strong base. The use of silver perchlorate as a catalyst is an alternative practical option when labelling alkynes which are sensitive to base or contain functionalities which would lead to labelling elsewhere in the molecule. Labelling with this catalyst takes place smoothly at ambient temperature in a mixture of *N*,*N*‐dimethylformamide and deuterium oxide.

## Graphical abstract

Terminally deuterated alkynes may be prepared by base‐catalysed isotope exchange with deuterium oxide, using sodium hydroxide, calcium oxide, or N,N,N,N‐tetramethylguanidine as catalysts. Silver perchlorate is a more suitable catalyst when labelling alkynes which are sensitive to base, or which contain functionalities which would lead to base‐catalysed labelling elsewhere in the molecule. The silver‐catalysed procedure is regiospecific and gives excellent yields and high deuterium abundances.

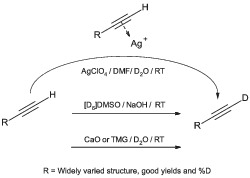

## 1 INTRODUCTION

**FIGURE 1:**
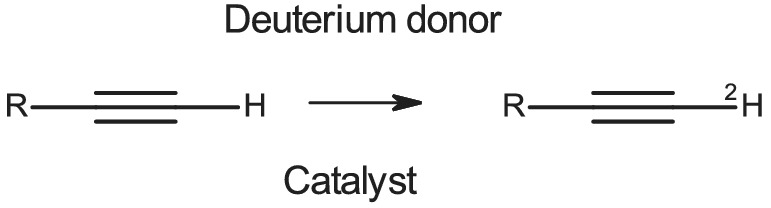
Labelling of terminal alkynes by catalytic isotope exchange

As part of our continuing studies^1^ of the isotopic exchange and reduction of alkenes over platinum group metals, we required a source of terminally labelled variously functionalised alkynes. Exchange labelling (Figure 1) seemed the simplest general approach.

Since the methine proton of terminal alkynes is acidic (pKa 26‐29), isotopic exchange with a suitable isotope donor in the presence of a strong base was frequently utilised, and this approach proved quite suitable for simple alkynes with no base‐sensitive functions. Four developments of this classical base‐catalysis approach have been published recently, one of which uses potassium carbonate^2^ as the base in acetonitrile/^2^H_2_O and another which employs a basic resin^3^ in admixture with toluene and ^2^H_2_O. Another methodology which uses base in conjunction with a ruthenium (II) pincer complex has also been described,^4^ whilst alkyne exchange catalysed by triethylamine has been utilised as one step in the preparation of specifically deuterated alkenes.^5^ Very recently, base‐catalysis with sodium hydroxide had been used to prepare terminally deuterated alkyne Raman probes for in situ tracking of small molecules and for measurement of physical chemistry parameters in live cells.^6^

The above methodologies provide routes to the labelled alkynes for those compounds stable to the conditions. However, since many functionalised alkynes are sensitive to basic conditions, we also sought an alternative mild, non‐basic, high‐yielding, and hence generally applicable procedure.

The complexation of alkynes with metals is well known. Such coordination can result in a significant change in alkyne bond polarisation, and possibly even in geometry, generating an increase in terminal proton acidity.^**7**^ This can lead to enhancements in isotopic exchange rates of more than 10^5^. We therefore studied metal‐catalysed deuterium exchange, with a range of alkynes and potential metal catalysts.

## 2 RESULTS AND DISCUSSION

In our hands, we found that base‐catalysed deuteration of alkynes was simple and efficient. The donor could be ^2^H_2_O, CH_3_O^2^H or [^2^H_6_]dimethylsulfoxide. The results of a simple NMR study using [^2^H_6_]DMSO over solid sodium hydroxide are shown in Table 1.

**TABLE 1:** Labelling of various terminal alkynes (0.5 mmol) in [^2^H_6_]dimethylsulfoxide (0.6 ml) over solid sodium hydroxide (100 mg pellet) for 24 h

Alkyne	% terminal ^2^H
Pentyne	95
Hexyne	>99
Phenylacetylene (1)	93
3‐Hydroxypentyne	>99
4‐Hydroxybutyne	>99
1‐Ethynylcyclohexanol (6)	96

Recently, this facile deuteration of alkynes using a [^2^H_6_]DMSO donor has been utilised as an initial step in the tandem deuterioamination, deuteriothiolation, deuteriophenoxylation, and deuterioalkoxylation of alkynes.^8^

Larger (millimolar or greater) scale preparative examples which used ^2^H_2_O as the isotope donor and sodium hydroxide, calcium oxide or *N*,*N*,*N*,*N*‐tetramethylguanidine as the basic catalyst are given in Section 4.

The results with various other bases and deuterium oxide are also summarised in Tables 2 and 3.

**TABLE 2:** Catalysis of phenylacetylene deuteration by various bases: phenylacetylene (1 mmol), tetrahydrofuran (0.9 ml), deuterium oxide (100 μl), time (4.5 h)

Base catalyst	Quantity (mmol)	%^2^H
Potassium carbonate	0.15	58
Sodium bicarbonate	0.15	5
Triethylamine	0.07	35
Calcium oxide	0.21	77
Magnesium carbonate	0.14	1
*N*,*N*,*N*,*N*‐Tetramethylguanidine	0.11	78

**TABLE 3:** Deuteration of eight alkynes by *N*,*N*,*N*,*N*‐tetramethylguanidine and calcium oxide catalysis: Substrate (1 mmol), THF (1.5 ml) deuterium oxide (1.4 ml) and either *N*,*N*,*N*,*N*‐tetramethylguanidine (0.15 mmol) or calcium oxide (0.36 mmol), stirred for 5 h at ambient temperature

	Catalyst	
Alkyne	*N,N,N,N*‐Tetramethylguanidine (TMG) (%^2^H, %yield)	Calcium oxide (%^2^H, %yield)
Phenylacetylene (1)	90, 78	90, 72
4‐Pentylphenylacetylene (3)	92, quant	92, quant
Propargyl benzoate (7)	91, 81	91, quant
Propargyl 4‐nitrobenzoate (8)	90, quant	92, 74 (97, 66)
Propargyl *N*‐phenylcarbamate (18)	88, 97	89, 99
1,1‐Diphenyl‐prop‐2‐yn‐1‐ol (13)	84, quant	73, quant
Dipropargyl terephthalate (9)	88, quant	Hydrolysed
1‐Ethynylcyclohexan‐1‐ol (6)	83, quant	86, 99

Potassium carbonate^2^ and triethylamine^5^ have previously been identified as a suitable bases; hence, our more recent studies were limited to the other two effective catalysts, calcium oxide and *N,N,N,N*‐tetramethylguanidine. Table 3 lists their efficacy and limitations.

The results show good labelling and excellent recoveries for most of the alkynes tested. The low recovery for propargyl 4‐nitrobenzoate (8) arose from partial hydrolysis whilst dipropargyl terephthalate (9) yielded a mixture of partially and completely hydrolysed product with no detectable starting diester. The esters 8 and 9 were selected for testing because they are more easily hydrolysed than the simple benzoate ester (7). The results emphasise the limitation of base catalysis. Thus, carboxylic and sulphonic esters may hydrolyse, tertiary acetylenic alcohols may undergo elimination whilst propargylic carbamates can cyclise^9^ to alkylidene oxazolidinones under basic conditions. Under the non‐forcing conditions employed in Table 3, however, the recovery of labelled propargyl *N*‐phenylcarbamate (18) and 1‐ethynlycyclohexan‐1‐ol (6) was good.

An additional complication of base catalysis arises for functional groups containing exchangeable protons, for example, alkylketones, alkylsulfoxides, or other compounds with activated methyl, methylene, or methine functions, where basic conditions could give rise to facile competitive labelling at the activated sites. Hence, another method not involving basic conditions was of interest.

An alternative approach to base‐catalysis is to utilise catalytic metals. Hence, more than 10 years ago, we began our search for an alternative metal catalyst by screening materials already known to be active in the exchange of terminal alkyne protons, particularly those containing palladium, silver, and copper. Initially, these GC‐MS screens employed a simple model alkyne substrate: phenylacetylene (ethynylbenzene, 1).

Those catalysts which were identified as having some activity in the GC‐MS screens are shown in Table 4. Subsequently deuterium NMR studies showed that the silver(I) catalysis was completely regiospecific for terminal exchange, and hence, silver was selected for more extensive studies.

**TABLE 4:** Screening of potential metal‐based catalysts for labelling phenylacetylene using silver(I) trifluoromethylsulphonate in tetrahydrofuran/CH_3_O^2^H

Catalyst	%^2^H
Silver(I) perchlorate	86
bis (Copper(I)triflate).benzene	79
Silver(I) tetrafluoroborate	77
Silver(I) trifluoromethanesulphonate	72
Silver(I) hexafluorophosphate	72
5% Palladium on CaCO_3_	56
5% Palladium on BaSO_4_	16
5% Palladium on carbon	11
Silver(I) acetate	9
Silver(I) oxide	5

There were clear literature precedents for silver ion catalysis, but these earlier investigations consisted mainly of in situ NMR studies of reaction rate and were designed primarily to investigate C–H activation mechanisms. They were not intended to provide preparative methodology. The literature reactions were carried out in [^2^H_3_]nitromethane (a solvent which stabilises Ag^+^ π‐complexes), and the authors used [^2^H_4_]acetic acid as the deuterium donor.^**7**^ Moreover, stoichiometric, or even greater, amounts of catalyst were used to facilitate the kinetic studies, as turnover was quite low. Hence, these literature systems were impractical for preparative deuteration purposes, and completely unsuitable for tritium‐labelling. We therefore studied the exchange reaction with CH_3_O^2^H or ^2^H_2_O donors (for deuterium‐labelling applications) and with a ^2^H_2_O donor as a model for a typical tritium‐labelling procedure. In the course of these studies, we identified Ag^+^ in conjunction with a non‐coordinating counter ion as an excellent deuteration catalyst.^10^ Subsequently, we went on to publish a full experimental description of a silver(I)‐catalysed labelling procedure for the regiospecific deuteration of terminal alkynes using AgClO_4_ as catalyst and ^2^H_2_O as donor.^11^

**FIGURE 2:**
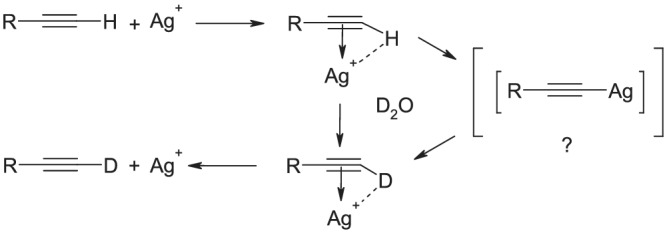
Proposed catalytic mechanism (see reference Yamada et al.^3^)

A proposed mechanism for the exchange process, possibly involving a 3‐centre bond, has been advanced (Figure 2).^7^, ^12^ As silver acetylide formation is generally very slow in the absence of base, it is likely that the majority of the exchange takes place via the π‐complex. Nevertheless, the potential presence of small amounts of the acetylide salt places restrictions on the work‐up procedure, necessitating that the initial deactivation of the catalyst should utilise a deuterated medium to ensure very high atom% deuteration.

Our studies showed high catalytic activity only for those silver salts with non‐complexing or weakly‐pairing counterions, suggesting that the catalytic species in the mechanism was essentially the uncomplexed Ag+ ion.

We subsequently concentrated on the four most active and water‐soluble silver catalysts of this type: AgBF_4_, AgO_3_SCF_3_, AgPF_6_, and AgClO_4_, all of which had similar activity.

To use the approach for labelling lipophilic organic compounds clearly required a co‐solvent, hence a range of aprotic solvents were studied using AgO_3_SCF_3_ as catalyst under comparative (i.e., non‐equilibrium) conditions (Table 5). This revealed that both *N*,*N*‐dimethylformamide and *N*‐methylpyrrolidinone yielded highest deuterium incorporation and these solvents were therefore employed for our later studies.

**TABLE 5:** Effect of co‐solvent on the deuterium labelling of phenylacetylene

Co‐solvent	%^2^H
Tetrahydrofuran	32
Dimethylsulphoxide	39
*N,N*‐Dimethylformamide	47
Acetone	41
*N,N*‐Dimethylacetamide	9
*N*‐Methyl‐2‐pyrrolidinone	53
Acetonitrile	32

**FIGURE 3:**
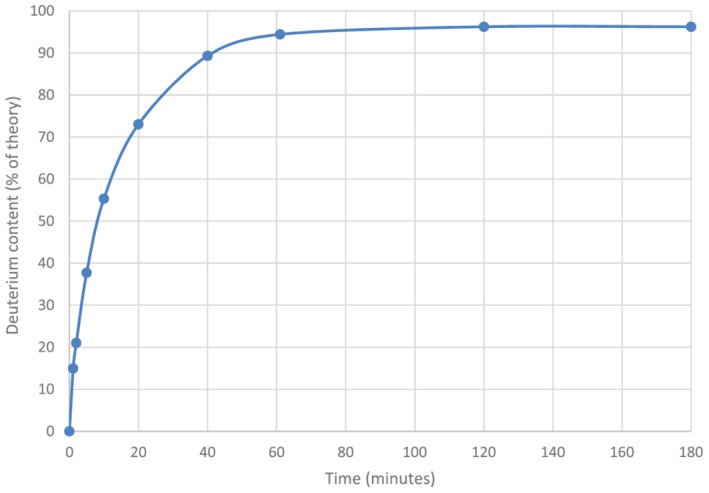
Rapid labelling of phenylacetylene (1 ml) using, AgClO_4_ catalyst (175 mg) in *N*,*N*‐dimethylformamide (12 ml) and ^2^H_2_O (1.2 ml) at room temperature

Our final optimised labelling procedure,^11^ which was shown to be suitable for labelling an easily hydrolysed alkyne diester,^13^ utilised silver perchlorate as the catalyst of choice. This salt is easy to handle and, although it is quite hygroscopic, in our hands, it was less so than the deliquescent triflate, tetrafluoroborate, and hexafluorophosphate salts. This obviated the need for strict handling in a glove box. Moreover, the salt is freely soluble in organic solvents. Of the two dipolar aprotics, we generally utilise DMF as the co‐solvent, since with some alkynes, reactions in *N*‐methylpyrrolidinone show progressive precipitation of a white solid making kinetic studies difficult. The optimised procedure is rapid (Figure 3) with a typical initial turnover frequency (calculated from initial rate data as mol of alkyne deuterated per mol of silver perchlorate catalyst employed, per minute of reaction) at room temperature of approximately 1 min^−1^ for a range of alkynes. In general, the reactions proceed smoothly at room temperature and are essentially quantitative, with no by‐products observable by GC‐MS.

**FIGURE 4:**
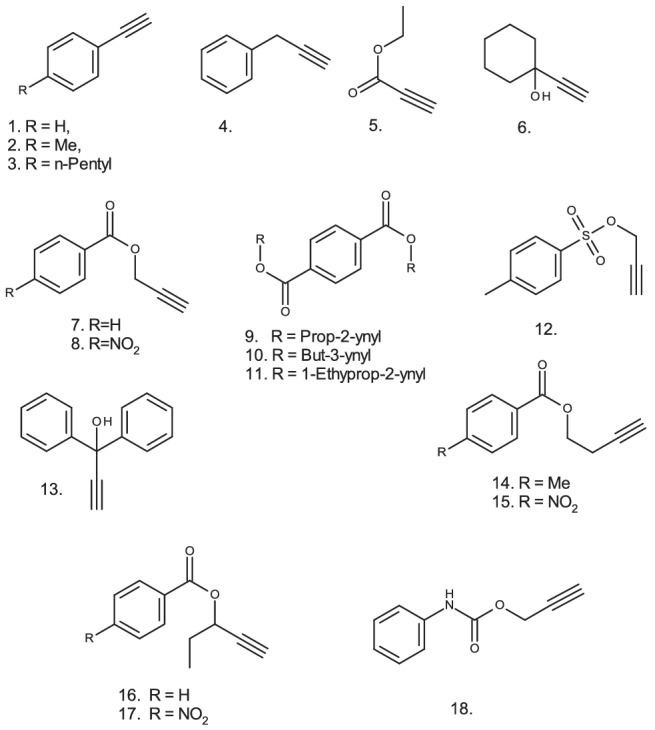
Structures of 18 alkynes labelled in the terminal position using silver perchlorate catalyst in dimethylformamide/deuterium oxide at RT

Moreover, the procedure has proved applicable to a range of alkynes of varied structure (Figure 4) including easily hydrolysed esters, tosylates, and alkynols activated towards elimination. Three examples of the use of the procedure for the preparation of terminally deuterated alkynyl esters (including two easily hydrolysed substrates) are given in Section 4. The reactions were carried out on the millimolar scale with good recovery and with good isotopic abundance in a single exchange step.

The potential specificity of the methodology for terminal labelling is one advantage of this procedure. Since the system is non‐basic, it should not introduce deuterium at alternative molecular sites such as the sites α‐to a ketone or sulfoxide or sites otherwise activated towards exchange under basic conditions, such as alkyl‐substituted nitroaromatics.^14^, ^15^ To check this, two such substrates, 1,3‐diacetylbenzene and 2,4‐dinitrotoluene, were investigated under standard terminal alkyne labelling conditions with *N,N,N,N*‐tetramethylguanidine, calcium oxide and silver perchlorate as catalysts. In the case of silver perchlorate no labelling whatsoever was observed for either substrate, whilst the two basic catalysis systems yielded [^2^H_6_‐dimethyl]diacetylbenzene and [^2^H_3_‐methyl]dinitrotoluene, confirming this advantage of the silver perchlorate system.

**FIGURE 5:**
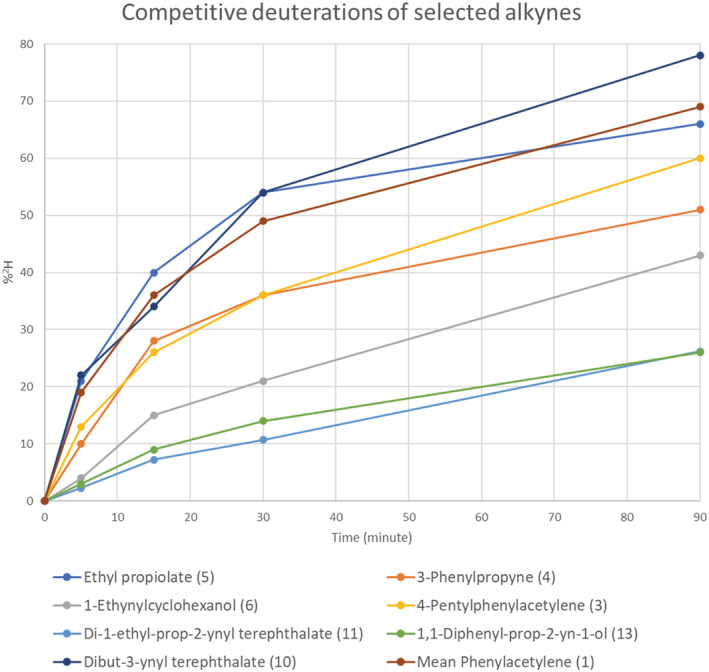
Deuteration of selected alkynes in comparison with an in‐situ phenylacetylene standard. The alkynes (0.25 mmol) were deuterated in *N,N‐*dimethylformamide (1 ml) containing deuterium oxide (4.6 mmol) and silver perchlorate (0.03 mmol)

To confirm the generality of the procedure and to investigate any obvious stereoelectronic effects, batches of alkynes of varied structure were selected and reacted under competitive conditions. The alkynes were then separated and analysed by GCMS to determine their deuterium content with increasing reaction time. Phenylacetylene was included in each batch of compounds as a standard. The results are collected in Figure 5 where the extent of deuteration of seven alkynes (in comparison with that of the phenylacetylene standard) is shown against reaction time.

The results show that most substrates deuterate at broadly similar rates, suggesting that the methodology will have wide applicability. However, there is an indication of steric effects, as the three most hindered alkynes (6, 11, and 13) underwent the slowest deuteration. Of course, these are competitive reactions, and such effects may not be of practical significance when a single alkyne is undergoing deuteration.

Kinetic isotope effects have been determined for several substrates in a related system which utilises silver triflate as catalyst and [^2^H_4_]acetic acid as the deuterium donor.^12^ These studies show that the reaction displays an *inverse* isotope effect (with the result that dedeuteration is more rapid than deuteration). The authors ascribed this behaviour to the change in hybridisation of the terminal sp carbon to an approximately sp^2^ state during the exchange process, or possibly to a 3‐centre agostic interaction which preferentially stabilises the C‐^2^H bond in the reaction transition state. Whichever is the case, such an isotope effect must therefore reflect the inherent structural, energetic or electronic differences between the ^1^H‐ and ^2^H‐alkyne Ag^+^ transition states. Hence, it is unsurprising that isotope effects were also observed in our exchange studies. Under equilibrium conditions the zero‐point energy term accounts for almost all of the isotope effect, resulting in the heavier isotope preferentially occupying the bond with the highest vibrational frequency.^16^, ^17^ In this case, that is the O–H bond in preference to the lower frequency alkyne C–H bond.

We studied the position of isotopic equilibrium for three alkyne substrates (1, 4, and 13) when the isotopic donor was a 1:1 molar ratio of ^1^H_2_O:^2^H_2_O used in excess, and found ^1^H:^2^H ratios at equilibrium of 1.1, 1.2 and 1.3:1. Previously, Lewandos et al.^12^ found values from 1.4, 1.6, 1.6, and 1.7:1 for the ratios of ≡‐^1^H:≡‐^2^H for four aliphatic alkynes using their silver triflate/deuteroacetic acid/deuteronitromethane system. This suggests that a similar type of exchange process may well be occurring in both systems.

Of course, for deuterium‐labelling purposes, even in the presence of this isotope effect, a suitable molar excess of deuterium oxide can be used to improve the atom% abundance achieved. Thus, propargyl benzoate can be prepared in good yield and at 96.6% deuterium content in a single exchange cycle by increasing the quantity of ^2^H_2_O used, as described in Section 4. Similarly, the more activated difunctional ester dipropargyl terephthalate is recovered, in essentially quantitative yield, with a deuterium abundance of 97.1% deuterium. As with phenylacetylene and 4‐pentylethynylbenzene, both these products showed only a single terminal deuteroalkyne C‐^2^H peak in the ^2^H‐NMR.

However, for tritium‐labelling, the methodology could be somewhat more problematic since the equilibrium isotope effect is likely to be greater for tritium. It should be borne in mind, therefore, that tritiation via this approach could yield somewhat lower specific activities than that of the tritium donor. For labelling at low specific activities, the specific activity of the donor tritiated water utilised would therefore need to be raised to allow for the isotope effect. Moreover, for tritium labelling any loss of label, due to back‐exchange during work up, or arising from the hydrolysis any acetylide present, could not be mitigated by the use of tritiated reagents.

When a simple model reaction, using deuterium oxide rather than tritium oxide, was carried out, and a small fraction (corresponding to the scale of a small no‐carrier‐added tritiation reaction) was worked‐up with unlabelled hydrochloric acid the product still showed approximately 76% of the theoretical labelling. In subsequent larger scale reactions, it proved possible to obtain higher deuterium levels by termination of the reaction with sodium chloride solution (85% ^2^H) or benzyltriethylammonium chloride (90% ^2^H). Hence, these reagents may prove better suited to termination of a tritiation reaction than hydrochloric acid, which gave only 74% ^2^H in the same study.

The use of an appropriate termination reagent and an excess of tritiated water should therefore ensure that the exchange labelled alkyne has a substantial fraction of the theoretical specific radioactivity. However, it should be borne in mind that alkynes are radiosensitive and hence the prediction of yield or specific activity from a deuterium model is open to error.

Since this work was carried out, a similar Ag^+^‐catalysed procedure for the preparation of terminally deuterated alkynes has been published.^18^ In that procedure, deuteration was achieved using deuterium oxide as donor and silver trifluoroacetate as catalyst in a dichloromethane/deuterium oxide mixture. The procedure gives good recoveries and deuterium abundances for a range of propargyl ethers, propargylamines, and phenylacetylene analogues and could form an effective alternative to our procedure for those alkynes suited to the reaction conditions.

One safety point to note is that AgClO_4_ is an oxidising agent, whilst alkynes themselves are easily oxidised species. Hence, care should be taken during reaction work‐up to ensure complete removal of the perchlorate (or of any released perchlorates or perchloric acid) before distillation or other thermal treatment. If this cannot be achieved, but such procedures are nevertheless required, then the silver perchlorate could be replaced with the triflate or tetrafluoroborate which have similar catalytic activity.

## 3 CONCLUSIONS

Base‐catalysed isotope exchange is a viable procedure for the preparation of terminally‐deuterated alkynes for those alkynes which are stable to basic conditions. [^2^H_6_]Dimethylsulfoxide is suitable as a deuterium donor when used in conjunction with solid sodium hydroxide as base. For exchange reactions employing deuterium oxide as donor, calcium oxide or *N*,*N*,*N*,*N*‐tetramethylguanidine are suitable base‐catalysts.

The use of Ag^+^‐catalysed terminal alkyne exchange is an alternative practical option when labelling alkynes which are sensitive to base, or which contain functionalities which would lead to base‐catalysed labelling elsewhere in the molecule. Silver perchlorate is a suitable catalyst for labelling at ambient temperature, and DMF or NMP are suitable co‐solvents. The labelling reaction demonstrates an equilibrium isotope effect which could reduce the isotopic abundance achieved unless a suitable excess of isotope donor is employed. Although this poses little problem for deuteration or intermediate specific activity tritium labelling, it could be significant for high specific activity labelling.

## 4 EXPERIMENTAL

### 4.1 Reagents

Alkynes were purchased from Merck (Sigma‐Aldrich Company Ltd), The Old Brickyard, New Road, Gillingham, Dorset, SP8 4XT, UK, or were prepared by acylation of the appropriate alkynol with the corresponding acyl or sulphonyl chloride, using triethylamine as base, in dichloromethane. Deuterium oxide (99.9 atom%^2^H) and *N*,*N*‐dimethylformamide, DMF, (<0.005% water content) were also obtained from Merck (address above). General solvents and reagents were obtained from regular chemical supply houses and were used as received.

### 4.2 Equipment

^1^H‐NMR spectra were recorded on Bruker spectrometers in C^2^HCl_3_ at 500 MHz or 300 MHz for ^1^H and at 126 MHz or 100 MHz for ^13^C. ^2^H‐NMR spectra were recorded in C^1^HCl_3_ at 77 MHz or 61 MHz. All spectra were analysed by Bruker TopSpin software. Raman spectra were measured on crystalline samples or on the neat liquid supported on glass microscope slides using a ThermoScientific DXR Raman Microscope at laser frequencies of either 532 nm or 780 nm. Infrared spectra were recorded for crystalline or neat liquid samples on a sapphire anvil using an Agilent Resolutions Pro spectrometer. GC‐MS analysis was performed using 1‐μl injections of solutions at approximately 1 mg/ml in dichloromethane using an Agilent 6890N gas chromatograph coupled to a 5973 mass selective detector. The column was a Phenomenex ZB‐5MS, 30 m × 0.25 mm, used with a temperature profile: 50°C (held for 3 min), ramp at 10°C per minute to 250°C and held for 2 min. The carrier gas was helium at 1 ml/min. The molecular formulae for the labelled compounds were determined at the National Mass Spectroscopy Facility, University of Swansea, using an LTQ Orbitrap XL operating in APCI or NSI modes.

### 4.3 Typical base catalysis procedures using sodium hydroxide in deuterium oxide

#### 4.3.1 [1‐^2^H]1‐Hexyne

1‐Hexyne (4.8 g, 58.4 mmol) was added to ^2^H_2_O (20 ml, 1 mol) containing four pellets (360 mg, 9 mmol) of sodium hydroxide and the biphasic mixture stirred thoroughly, ensuring a vortex to mix the layers, for 4.5 h at room temperature. NMR of a small aliquot after this time showed approximately 95–96% deuteration. The 1‐hexyne layer was allowed to separate overnight then removed with a Pasteur. It was then washed with ^2^H_2_O (4 ml) then with potassium dihydrogen phosphate (100 mg/ml in ^2^H_2_O, 2 ml) and finally twice with ^2^H_2_O (2 ml). The resulting product was dried with anhydrous MgSO_4_ for 3 h and then filtered. Yield 3.7 g (76%). Pure by NMR, ^2^H content 95.5%. Losses were due to the reaction scale and to volatility during the work‐up. ^1^H‐NMR, δ (C^2^HCl_3_, 300 MHz) 0.92 (t, J = approximately 7.5 Hz, 3H), 1.41 (complex multiplet, J = approximately 7.5 Hz, 2H), 1.52 (very complex multiplet, J = approximately 7.5H, 2H), 1.92 (trace of residual alkyne proton, J = approximately 2.8 Hz, 0.04‐0.05H), 2.19 (t, J = approximately 7.4 Hz, 2H) ppm. ^2^H‐NMR, δ (CHCl_3_, 77 MHz) singlet 1.9 ppm. No other resonances. ^13^C‐NMR, δ (C^2^HCl_3_, 126 MHz) 13.6, 18.2, 21.9, 30.7, 68.0 (v. weak 1:1:1 triplet, J = 38 Hz, C‐^2^H alpha), 84.5 (v. weak 1:1:1 triplet, J = 8 Hz, C‐^2^H beta).

The alkynes below were prepared by a similar procedure.

#### 4.3.2 [1‐^2^H]1‐Pentyne

1‐Pentyne (3.98 g, 58.4 mmol) was added to ^2^H_2_O (20 ml, 1 mol) containing sodium hydroxide (400 mg, 10 mmol), and the biphasic mixture stirred for 4.5 h at room temperature. The aqueous layer was removed and replaced with ^2^H_2_O (10 ml) containing sodium hydroxide (200 mg), and the reaction stirred for a further 2.5 h, The 1‐pentyne layer was allowed to separate, removed with a Pasteur pipette, washed with ^2^H_2_O (4 ml), then with potassium dihydrogen phosphate (100 mg/ml in ^2^H_2_O, 2 ml) and dried with anhydrous MgSO_4_. Yield 2.1 g, 52% (work up losses were due to volatility). 98%^2^H. ^1^H‐NMR, δ (C^2^HCl_3_, 300 MHz) 1.00 (t, J = approximately 7.5 Hz, 3H), 1.55 (hexuplet, J = approximately 7.5 Hz, 2H), 1.95 (t, J = approximately 2.8 Hz, small trace of residual alkyne), 2.17 (t, J = approximately 7.4 Hz, 2H) ppm.

#### 4.3.3 [1‐^2^H]3‐Phenylprop‐1‐yne

3‐Phenylprop‐1‐yne (0.68 g, 5.85 mmol) was deuterated on 1/10th the above scale of the 1‐pentyne procedure. Yield 59%, approximately 98.5%^2^H. m/z 117, 116, 115, 89, 88, 74, 63, 51. ^1^H‐NMR, δ (C^2^HCl_3_, 300 MHz), 2.3 (trace of residual alkyne proton, approximately 0.01H), 3.7 (s) 2H, 7.3–7.5 (very complex multiplet) 5H ppm.

#### 4.3.4 [2′‐^2^H]Phenylacetylene

Phenylacetylene (1.2 g, 11.75 mmol) was deuterated on approximately 1/5th the scale. Yield 75%, 98%^2^H. m/z 103, 86, 77, 76, 63, 51. ^1^H‐NMR, δ (C^2^HCl_3_, 300 MHz), 3.1 s (trace of residual alkyne proton, approximately 0.02H), 7.38 (cm) 3H, 7.58 (cm) 2H ppm.

### 4.4 Typical base catalysis procedure using calcium oxide

#### 4.4.1 4‐Pentyl‐[2′‐^2^H]ethynylbenzene

4‐Pentyl‐ethynylbenzene (172 mg, 1 mmol) was dissolved in tetrahydrofuran (1.5 ml) and added to calcium oxide (60 mg, 1.07 mmol) and deuterium oxide (1.5 ml, 75 mmol) added. The reaction mixture was stirred at room temperature for 24 h before work up. Acetic anhydride (120 μl, 1.26 mmol) was allowed to react with deuterium oxide (400 μl, 20 mmol) for 0.5 h (until homogeneous) to produce deuterated acetic acid and the mixture added to the reaction and stirring continued for 10 min. Dichloromethane (3 ml) was added, the mixture shaken, and the dichloromethane layer allowed to separate and then washed with water (0.5 ml) three times, filtered, dried over anhydrous magnesium sulphate, the filter cake washed twice through with dichloromethane (2 ml) and the combined filtrates evaporated to yield 4‐pentyl‐[2′‐^2^H]ethynylbenzene (155 mg, 90%, 96.3%^2^H): 173 (M), 129, 116, 103, 90, 89, 76, 63, 51, ^1^H‐NMR, δ (C^2^HCl_3_, 500 MHz), 0.87 (t, J = approximately 7.0 Hz, 3H), 1.30 (complex multiplet, 4H), 1.59 (pentuplet, J = approximately 7.5 Hz, 2H), 2.59 (t, J = approximately 7.5 Hz, 2H), 3.01 (small trace of residual alkyne proton), 7.12 (d, J = approximately 8 Hz, 2H), 7.39 (d, J = approximately 8 Hz, 2H) ppm; ^2^H‐NMR, δ (C^1^HCl3, 77 MHz) 3.00 ppm, singlet, no other resonances; ^13^C‐NMR, δ (C^2^HCl_3_, 126 MHz**)** 14.0, 22.3, 31.3, 31.4, 35.8, 76.5 (small multiplet, partially obscured by the C^2^HCl_3_ resonance, J = approximately 37 Hz), C‐^2^H), 83.6, 119.3, 128.4,132.0, 144.0 ppm; ʋ_max_ 3028, 2928, 2956, 2587 (strong, ≡C‐^2^H), 1607, 1507, 1466, 1420, 1400, 1110, 1010, 839, 816, 736 cm^−1^.

### 4.5 Typical base catalysis using *N*,*N*,*N*,*N*‐tetramethylguanidine

#### 4.5.1 [3‐^2^H]Propargyl benzoate

Propargyl benzoate (155 mg, 0.97 mmol) was dissolved in tetrahydrofuran (1.5 ml) containing *N*,*N*,*N*,*N*‐tetramethylguanidine (30 mg, 0.26 mmol) and then deuterium oxide (1.5 ml, 75 mmol) added. The reaction mixture was stirred at room temperature for 24 h before work up. Acetic anhydride (120 μl, 1.26 mmol) was allowed to react with deuterium oxide (400 μl, 20 mmol) for 0.5 h (until homogeneous) to produce deuterated acetic and the mixture added to the reaction. Dichloromethane (3 ml) was added, the mixture shaken, and the dichloromethane layer allowed to separate and then washed with water (0.5 ml) three times, filtered, dried over anhydrous magnesium sulphate, the filter cake washed twice through with dichloromethane (2 ml) and the filtrate evaporated to yield [3‐^2^H]propargyl benzoate (124 mg, 80%, 95.7%^2^H): 161 (M), 132, 116, 105, 77, 51 amu, ^1^H‐NMR, δ (C^2^HCl_3_, 500 MHz), 2.52 (t, very small trace of residual alkyne proton), 4.92 (s) 2H), 7.45 (t, J = approximately 8HJz, 2H), 7.58 (t, J = approximately 8 Hz, 1H), 8.05 (d, J = approximately 8 Hz, 2H) ppm; ^2^H‐NMR, δ (CHCl_3_, 77 MHz) 2.5 ppm, singlet, no other resonances; ^13^C‐NMR, δ (C^2^HCl_3_, 126 MHz) 52.5, 74.8 (small 1:1:1 triplet, J = approximately 37 Hz, ^13^C‐^2^H), 75.2 (s, very small trace of undeuterated compound), 128.7. 129.5, 130.2, 133.1, 165.8 ppm.; ʋ_max_ 3040, 3015, 2920, 2593 (≡C‐^2^H), 1720, 1601, 1451, 1369, 1263, 1105, 1069, 1026, 974, 805, 700 cm^\−1^; Raman (532‐nm excitation) 3070, 2946, 2126, 1986, 1722, 1598, 1366, 1266, 1023, 999, 805, 613, 326 cm^−1^.

### 4.6 Typical neutral procedure using silver ion catalysis

#### 4.6.1 [3‐^2^H]Propargyl benzoate. (3‐deuterioprop‐2‐ynyl benzoate)

Propargyl benzoate (160 mg, 1 mmol) was dissolved in dry *N*,*N*‐dimethylformamide (2 ml) in a dry 10‐ml capacity polypropylene vial and a solution of silver perchlorate (19.5 mg, 0.094 mmol) in deuterium oxide (1 ml) added. The resulting cloudy solution was left at room temperature with occasional shaking for 24 h to ensure complete equilibration. The solution was then treated with deuterochloric acid (35% solution in ^2^H_2_O, 167 μl) diluted with deuterium oxide (834 μl, 41.7 mmol,). After standing for 2 min (to allow complete precipitation of silver chloride), dichloromethane (4 ml) was added, the mixture shaken, and the dichloromethane layer separated. The layer was washed twice with deuterium oxide (2 × 0.5 ml). The layer was then dried by passing through a 5‐cm column of anhydrous magnesium sulphate in a Pasteur pipette, washing through with a further portion (1 ml) of dichloromethane. Removal of the solvent by evaporation yielded [3‐^2^H]propargyl benzoate (134 mg, 83%, 96.6% ^2^H: 161 (M), 132, 116, 105, 77, 51 amu); C_10_H_7_^2^HO_2_Na (M + Na) requires 184.0479, found 184.0480 amu; ^1^H‐NMR, δ (C^2^HCl_3_, 500 MHz), 2.52 (trace of residual alkyne proton), 4.93 (2H), 7.45 (t, J = approximately 8 Hz, 2H), 7.58 (t, J = 8 Hz, 1H), 8.07 (d, J = approximately 8 Hz, 2H) ppm; ^2^H‐NMR, δ (CHCl3) 2.49 ppm, singlet, no other resonances; ^13^C‐NMR, δ (C^2^HCl_3_, 126 MHz), 52.4, 74.9 (small 1:1:1 triplet, J = approximately 36 Hz, ^13^C‐^2^H), 128.5, 129.4, 129.8, 133.3, 165.8 ppm.; ʋ_max_ 3064, 2949, 2869, 2592 (≡C‐^2^H), 1720, 1601, 1452, 1369, 1262, 1095, 1069. 1026, 974, 708 cm^−1^.

#### 4.6.2 [3‐^2^H]propargyl 4‐nitrobenzoate (3‐deuterioprop‐2‐ynyl 4‐nitrobenzoate)

Propargyl 4‐nitrobenzoate (205 mg, 1 mmol) was dissolved in dry *N.N*‐dimethylformamide (2 ml) in a dry 10‐ml capacity polypropylene vial and a solution of silver perchlorate (19.5 mg, 0.094 mmol) in deuterium oxide (1 ml) added. The resulting cloudy solution was stirred at room temperature for 24 h to ensure complete equilibration. The solution was then treated with deuterochloric acid (35% solution in ^2^H_2_O, 167 μl) diluted with deuterium oxide (834 μL, 41.7 mmol). After standing for 2 min (to allow complete precipitation of silver chloride), dichloromethane (4 ml) was added, the mixture shaken, and the dichloromethane layer separated. The layer was washed twice with deuterium oxide (2 × 0.5 ml). The layer was then dried with anhydrous magnesium sulphate and filtered, and the filter cake washed through with a further portion (1 ml) of dichloromethane. Removal of the solvent by evaporation yielded [3‐^2^H]propargyl 4‐nitrobenzoate (197 mg, 96%, 96.3%^2^H): 206 (M), 150, 120, 104, 92, 76 amu; C_10_H_7_^2^HO_4_N (M + H) requires 207.0511, found 207.0506 amu; ^1^H‐NMR, δ (C^2^HCl_3_, 500 MHz), 2.57 (trace of residual alkyne proton), 4.98 (2H), 8.26 (dt, J_*ortho*_ = 8.6 Hz, J_*meta*_ = 3.9 Hz, 2H), 8.30 (dt, J_*ortho*_ = 8.6 Hz, J_*meta*_ = 3.9 Hz, 2H) ppm; ^2^H‐NMR, δ (C^1^HCl_3_) 2.56 singlet, no other resonances; ^13^C‐NMR, δ (C^2^HCl_3_, 126 MHz), 163.9, 150.8, 134.8, 131.0, 123.6, 76.5, 75.5 (small 1:1:1 triplet, J = approximately 35 Hz, ^13^C‐^2^H), 53.3 ppm; ʋ_max_ 2920, 2840, 2588 (≡C‐^2^H), 1720, 1655, 1607, 1522, 1491, 1441, 1348, 1266, 1236, 1103, 1052, 954, 873, 832, 713 cm^−1^; Raman (532 nm excitation) 3097, 2975, 2947,1991,1717, 1597, 1520, 1366, 1345, 1099, 954, 861, 780, 626, 506, 300, 265 cm^−1^.

#### 4.6.3 [3,3′‐^2^H_2_]dipropargyl terephthalate (bis(3‐deuterioprop‐2‐ynyl) benzene‐1,4‐dicarboxylate)

Dipropargyl terephthalate (121 mg, 0.5 mmol) was dissolved in dry *N.N*‐dimethylformamide (2 ml) and ethyl acetate (2 ml) in a dry 10‐ml capacity polypropylene vial and a solution of silver perchlorate (19.5 mg, 0.094 mmol) in deuterium oxide (1 ml, 50 mmol) added. The resulting cloudy solution was left at room temperature with occasional shaking for 48 h to ensure complete equilibration. The solution was then treated with deuterochloric acid (35% solution in ^2^H_2_O, 167 μl) diluted with deuterium oxide (834 μl, 41.7 mmol). After standing for 2 min (to allow complete precipitation of silver chloride), dichloromethane (4 ml) was added, the mixture shaken, and the dichloromethane layer separated. The layer was washed twice with deuterium oxide (2 × 0.5 ml). The layer was then dried by passing through a 5‐cm column of anhydrous magnesium sulphate in a Pasteur pipette, washing through with a further portion (1 ml) of dichloromethane. Removal of the solvent by evaporation yielded [3,3′‐^2^H_2_]dipropargyl terephthalate (118 mg, 97%, 97.1%^2^H): MS, 244 (M), 243, 188, 187, 160, 132, 104, 76 amu; C_14_H_8_^2^H_2_O_4_Na (M + Na) requires 267.0597, found 267.0599 amu; ^1^H‐NMR, δ (C^2^HCl_3_, 500 MHz), 2.55 (trace of residual alkyne proton), 4.95 (s, 4H), 8.16 (s, 4H) ppm; ^2^H‐NMR, δ (C^1^HCl_3_) 2.52 ppm, singlet, no other resonances; ^13^C‐NMR, δ (C^2^HCl_3_, 126 MHz), 52.9, 75.0 (small 1:1:1 triplet, J = approximately 32 Hz, ^13^C‐^2^H), 129.9, 133.5, 164.9 ppm.; ʋ_max_ 3064, 2939, 2569 (≡C‐^2^H), 1712, 1443, 1408, 1368, 1119, 1014, 957, 720 cm^−1^; Raman (532 nm excitation) 3083, 2973, 2937, 2870, 1985, 1719, 1611, 1365, 1282, 1112, 961, 854, 703, 631, 578, 507, 313, 247 cm^−1^.

#### 4.6.4 Competitive deuterations

The alkynes were deuterated in batches, such that all the alkynes in each batch were separable and analysable by GC‐MS. The batches were 1. Phenylacetylene, ethyl propiolate; 2. Phenylacetylene and 3‐phenylpropyne; 3. Phenylacetylene, 1‐ethynylcyclohexanol, 4‐pentylphenylacetylene, di(1‐ethylprop‐2‐ynyl) terephthalate; 4. Phenylacetylene, di (but‐3‐ynyl) terephthalate, 1‐ethynyl‐1,1‐diphenylmethanol.

Silver perchlorate (29 mg. 0.14 mmol) was dissolved in DMF (2 ml) and deuterium oxide (0.4 ml, 20 mmol) added. Aliquots (0.5 ml) of the solution were added to solutions of mixtures of phenylacetylene (0.25 mmol) plus the test alkynes (0.25 mmol of alkyne proton each) in DMF (0.5 ml) and the resulting reactions worked‐up and monitored as follows: Aliquots (100ul) of the reaction mixtures were added to deuterochloric acid in deuterium oxide (20 μl of 37% ^2^HCl in ^2^H_2_O, 80 μl, 4 mmol) and left until all the silver chloride had precipitated (approximately 1 min), then partitioned between water (2 ml) and dichloromethane (3 ml). The dichloromethane layer was washed with water until neutral (2 × 4 ml) then dried with anhydrous MgSO_4_ and filtered. Aliquots of the filtrate (600 μl) were made up to 2 ml with dichloromethane for GC‐MS analysis. Deuterium contents were calculated from the MS ion intensities using the IsoPat^2^ program.^19^

#### 4.6.5 Deuteration of 4‐pentylphenylacetylene on a scale appropriate to tritiation

A mixture containing silver perchlorate (5 mg, 24 μmol) and 4‐pentylphenylacetylene (86 mg, 0.5 mmol) in DMF (1 ml) and deuterium oxide (0.1 ml, 5 mmol) was allowed to react overnight at room temperature. To model a high specific activity reaction a small aliquot (10 μl) from this reaction, containing [^2^H]4‐pentylphenylacetylene (4.55 μmol) and deuterium oxide (45.5 μmol) was worked‐up using only protiated reagents. Thus, the aliquot was treated with 2 molar hydrochloric acid (100 μl), and the resulting mixture partitioned between water (2 ml) and dichloromethane (2 ml). The dichloromethane layer was then separated, washed with water (4 ml), dried over anhydrous magnesium sulphate, and filtered. Analysis of the solution by GC‐MS showed that the labelled product contained 76.3% of the theoretical amount of deuterium.

## Data Availability

Supporting data are available from the corresponding author upon reasonable request.
